# AT-RvD1 Modulates CCL-2 and CXCL-8 Production and NF-*κ*B, STAT-6, SOCS1, and SOCS3 Expression on Bronchial Epithelial Cells Stimulated with IL-4

**DOI:** 10.1155/2015/178369

**Published:** 2015-05-05

**Authors:** Jhony Robison de Oliveira, Daniely Cornélio Favarin, Sarah Cristina Sato Vaz Tanaka, Marly Aparecida Spadotto Balarin, David Nascimento Silva Teixeira, Bruce David Levy, Alexandre de Paula Rogério

**Affiliations:** ^1^Institute of Health Sciences, Department of Clinical Medicine, Laboratory of Experimental Immunopharmacology, Federal University of Triangulo Mineiro, Street Vigário Carlos 162, 38025-350 Uberaba, MG, Brazil; ^2^Institute of Biological and Natural Sciences, Department of Genetics, Federal University of Triangulo Mineiro, Uberaba, MG, Brazil; ^3^Institute of Health Sciences, Department of Clinical Medicine, Federal University of Triangulo Mineiro, Uberaba, MG, Brazil; ^4^Pulmonary and Critical Care Medicine Division, Department of Internal Medicine, Brigham and Women's Hospital and Harvard Medical School, Boston, MA 02115, USA

## Abstract

Bronchial epithelial cells represent the first line of defense against microorganisms and allergens in the airways and play an important role in chronic inflammatory processes such as asthma. In an experimental model, both RvD1 and AT-RvD1, lipid mediators of inflammation resolution, ameliorated some of the most important phenotypes of experimental asthma. Here, we extend these results and demonstrate the effect of AT-RvD1 on bronchial epithelial cells (BEAS-2B) stimulated with IL-4. AT-RvD1 (100 nM) decreased both CCL2 and CXCL-8 production, in part by decreasing STAT6 and NF-*κ*B pathways. Furthermore, the effects of AT-RvD1 were ALX/FRP2 receptor dependent, as the antagonist of this receptor (BOC1) reversed the inhibition of these chemokines by AT-RvD1. In addition, AT-RvD1 decreased SOCS1 and increased SOCS3 expression, which play important roles in Th1 and Th17 modulation, respectively. In conclusion, AT-RvD1 demonstrated significant effects on the IL-4-induced activation of bronchial epithelial cells and consequently the potential to modulate neutrophilic and eosinophilic airway inflammation in asthma. Taken together, these findings identify AT-RvD1 as a potential proresolving therapeutic agent for allergic responses in the airways.

## 1. Introduction

Asthma is an inflammatory disease of the airways characterized by the migration and accumulation of leukocytes, particularly eosinophils, mucus hypersecretion, and bronchial hyperreactivity. The pathophysiology of asthma is coordinated by the immune response of CD4^+^ T cells, specifically the Th2 phenotype. IL-4 is the major cytokine involved in the Th2 immune response. IL-4 uses Janus kinases (JAKs) to initiate the signaling cascade and activate signal transducer and activator of transcription 6 (STAT6), consequently modulating allergic airway inflammation in asthma and other diseases [1]. Most patients with asthma have symptoms that are readily controllable by standard asthma therapies, including *β*2-adrenergic agonists, low doses of inhaled corticosteroids, or leukotriene modifiers [2]. However, 5–10% of asthmatic individuals have poorly controlled disease with frequent exacerbations or symptoms that are refractory to current therapy [3]. Th1 and Th17 cells promote neutrophil recruitment and have been associated with both severe and steroid-resistant asthma [4].

Bronchial epithelial cells are involved in the homeostasis and coordination of immune responses in the airways and represent the first line of defense against microorganisms and allergens in the lungs [5, 6]. These cells express pattern recognition receptors, such as Toll-like receptors (TLR), and protease-activated receptors (PARs), which recognize microorganisms and allergens, respectively [7, 8]. The activation of these receptors on epithelial cells induces the production of chemokines and the expression of adhesion molecules and cytokines [9, 10] that can influence dendritic cell maturation, T cell differentiation, and airway inflammation modulation [11–14]. Bronchial epithelial cells also express the receptor for IL-4 (IL-4RA), and the activation of these cells by IL-4 induces, among other inflammatory parameters [15], the production of chemokines, for example, CCL2, CXCL-8, among others [7, 13, 14, 16, 17], which modulate leukocyte traffic and consequently airway inflammation in asthma.

During inflammation, the essential omega-3 fatty acid docosahexaenoic acid (DHA; C22:6) is available for enzymatic transformation into several anti-inflammatory and proresolving mediators, including the class of molecules termed resolvins [18]. Resolvin and its epimer, Aspirin-Triggered-Resolvin D1 (AT-RvD1, R configuration at carbon 17), are enzymatically derived from DHA and demonstrate anti-inflammatory and pro-resolving effects in several experimental models, including in the airways in acute lung injury [19] and experimental airway allergic inflammation induced by ovalbumin [20] in mice. In this study, we investigated the role of AT-RvD1 on bronchial epithelial cells stimulated with IL-4.

## 2. Materials and Methods

### 2.1. Bronchial Epithelial Cells

The human bronchial epithelial cell line BEAS-2B (ATCC, Rockville, MD) was cultured in Dulbecco's modified Eagle's medium (DMEM-F12/Gibco-Life Technologies, Carlsbad, CA, USA) supplemented with 10% fetal bovine serum (Gibco-Life Technologies) and 1% penicillin + streptomycin (Gibco-Life Technologies, Carlsbad, CA, USA) and incubated at 37°C in a humidified atmosphere with 5% CO_2_ and 95% ambient air.

### 2.2. Stimulus and Treatment

AT-RvD1 was donated by Dr. David Bruce Levy of the Harvard Medical School. BEAS-2B (4 × 10^4^ cell/mL) cells were cultivated in 96-well plates and treated with AT-RvD1 (1–100 nM) or vehicle (absolute alcohol) for 30 minutes prior to IL-4 (25 ng/mL) [17] stimulation. The use of BOC1 (10 *μ*M), an ALX receptor antagonist, followed the same experimental procedure described above but was added 15 min before treatment with AT-RvD1 [21].

### 2.3. CCL2 and CXCL-8 Production in the Supernatant of Cells Treated with AT-RvD1 to Chemokine Quantification

The supernatant was collected at 24 h after IL-4 stimulation, and the CCL2 and CXCL-8 concentrations were measured by enzyme-linked immunosorbent assays (ELISA) according to the manufacturers' instructions (BD Pharmingen, San Diego, CA, USA).

### 2.4. Expression of NF-*κ*B and STAT6 in Cells Treated with AT-RvD1

The effect of AT-RvD1 on the NF-*κ*B and STAT6 pathways was assessed by cytometry according to Cao et al. [22]. Briefly, 15 min after IL-4 stimulation, cells were fixed with prewarmed BD Cytofix Buffer (4% paraformaldehyde) for 10 min at 37°C. After centrifugation, the cells were permeabilized in ice-cold methanol for 30 min and then stained with mouse monoclonal antibodies against anti-NF-*κ*B (BD Biosciences Pharmingen, Phosflow, USA), anti-STAT6 (BD Biosciences Pharmingen, Phosflow, USA), or their corresponding mouse IgG2b isotype (BD Biosciences Pharmingen, Phosflow, USA) for 60 min followed by an FITC- or PE-conjugated goat anti-mouse IgG2b secondary antibody for another 45 min at 10°C in the dark. The cells were then washed, resuspended, and subjected to analysis. The expression of intracellular phosphorylated signaling molecules in 50,000 viable cells was analyzed by flow cytometry (FACSCalibur; BD Biosciences Pharmingen).

The results for phosphorylated NF-*κ*B and STAT6 are shown as a percentage of fluorescence and are expressed as the arithmetic mean.

### 2.5. SOCS1 and SOCS3 Expression

At 1 h after IL-4 stimulation, total RNA was extracted from cells using Pure Linkr RNA Mini Kit (Life Technologies, Carlsbad, CA, USA). cDNA was synthesized by reverse transcription (RT) from total RNA with SuperScript VILO MasterMix ((Invitrogen), Carlsbad, CA, USA) according to the manufacturer's instructions. Duplicate qPCR reactions were performed with primers for SOCS1 (Forward: 5′-TTTT TCGCCCTTAGCGTGA-3′, Reverse: 5′-AGCAGCTCGAAGAGGCAGTC-3′) and SOCS3 (Forward: 5′-TGAGCGCGGCTACAGCTT-3′, Reverse: 5′-TCCTTAATGTCACGCACGATTT-3′) and control GAPDH (Forward: 5′-CCACCCATGGCAAATTCC-3′, Reverse: 5′-TCGCTCCTGGAAGATGGTG-3′) (Life Technologies) using cDNA-specific TaqMan Gene Expression Assays with an ABI 7500 Fast Real-Time PCR System (Applied Biosystems). In each 5 *μ*L TaqMan reaction, cDNA (corresponding to 100 ng reverse transcribed RNA) was mixed with 0.25 *μ*L TaqMan Gene Expression Assay, 2.5 *μ*L TaqMan Universal PCR Master Mix (Applied Biosystems), and 1.25 *μ*L H_2_O. The PCR conditions were 95°C for 20 s, followed by 50 cycles at 95°C for 3 s, and 60°C for 30 s. Negative control reactions with no cDNA present and three interrun calibrator samples were included on each assay plate.

The Ct (cycle threshold) values for SOCS1 and SOCS3 mRNA were normalized to GAPDH to provide the delta Ct values. The relative mRNA expression was determined using the Livak method (the 2^−ΔΔCt^ method for real-time PCR) [23].

### 2.6. Statistical Analysis

The results were expressed as the mean ± standard error of the mean. An evaluation of the results was performed by an analysis of variance (ANOVA) followed by a Tukey post-test among the means using GraphPad PRISM (Version 6.0; GraphPad Software Inc., San Diego, CA, USA). *P* values less than 0.05 were considered statistically significant.

## 3. Results

### 3.1. AT-RvD1 Reduces the Concentration of Chemokines

**Figure 1:**
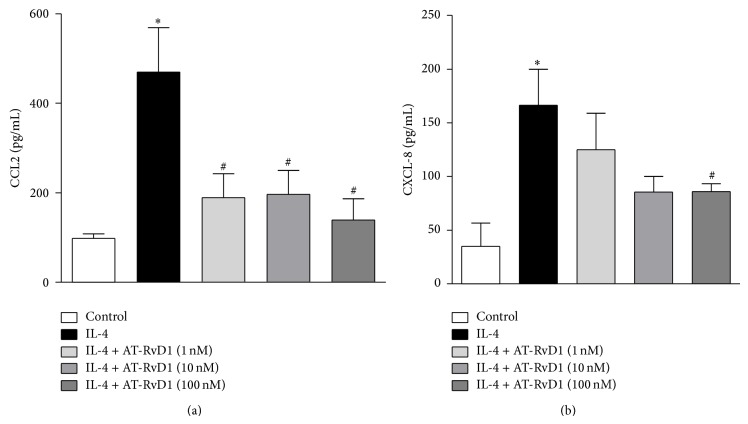
AT-RvD1 reduced the production of CCL2 (a) and CXCL-8 (b) in bronchial epithelial cells stimulated with IL-4. BEAS-2B cells were stimulated with IL-4 (25 ng/mL) in the presence or absence of AT-RvD1 (1–100 nM) for 24 h, and the culture supernatants were analyzed to determine CCL2 and CXCL-8 concentrations using an ELISA kit. The data are reported as the means ± SEM (*n* = 7). ^∗^ *P* < 0.05 versus control group; ^#^ *P* < 0.05 versus IL-4-treated group.

The activation of bronchial epithelial cells induces, among others, the release of chemokines [7, 13, 14, 16, 17]. Therefore, we evaluated the role of AT-RvD1 in CCL2 and CXCL-8 production in bronchial epithelial cells stimulated with IL-4. Our results showed that IL-4 stimulation (25 ng/mL for 24 h) induced a prominent increase in CCL2 and CXCL-8 concentrations compared to nonstimulated cells (control group; Figures 1(a) and 1(b), resp.). At all doses (1–100 nM), AT-RvD1 significantly reduced CCL-2 (Figure 1(a)) and CXCL-8 (Figure 1(b)) production when compared with the cells treated with IL-4, whereas no significant difference was observed in cells treated with vehicle compared to cells treated with IL-4 (data not shown).

### 3.2. The Inhibitory Effect of AT-RvD1 on Chemokine Production Is ALX/FPR2 Receptor Dependent

**Figure 2:**
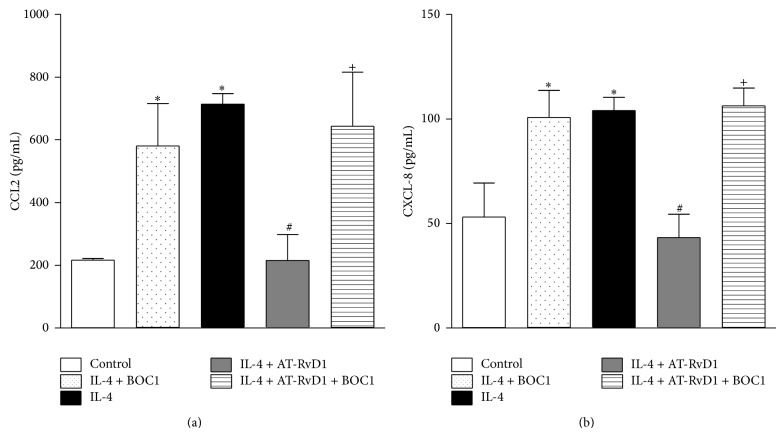
AT-RvD1 reduces CCL2 (a) and CXCL-8 (b) production in BEAS-2B cells stimulated with IL-4 through ALX/FPR2 receptor activation. BEAS-2B cells were stimulated with IL-4 (25 ng/mL) in the presence or absence of AT-RvD1 (100 nM) or in combination with BOC1, an ALX selective antagonist (10 *μ*M), for 24 h; the culture supernatants were analyzed for CCL2 and CXCL-8 concentrations using an ELISA kit. The data are reported as the means ± SEM (*n* = 7). ^∗^ *P* < 0.05 versus control group, ^#^ *P* < 0.05 versus IL-4-treated group, and ^+^ *P* < 0.05 versus IL-4 + AT-RvD1(100 nM) treated group.

The results presented above demonstrated that AT-RvD1 modulated the chemokine production induced by IL-4 in bronchial epithelial cells. Recent findings have shown that AT-RvD1 exerts part of its proresolving effects via interactions with the ALX/FPR2 receptor present on bronchial epithelial cells [24, 25]. Accordingly, we verified whether the ALX/FPR2-selective antagonist, BOC1, is capable of blocking the effects of AT-RvD1 on chemokine release by BEAS-2B cells after IL-4 stimulation. As demonstrated above, IL-4 stimulated CCL-2 and CXCL-8 production, and AT-RvD1 reduced both (Figures 2(a) and 2(b), resp.). Interestingly, BOC1 significantly reversed the inhibitory effect of AT-RvD1 on CCL2 (Figure 2(a)) and CXCL-8 (Figure 2(b)) production. No significant difference was observed in cells stimulated with IL-4 and treated with BOC1 (10 *μ*M) when compared with cells treated with IL-4.

### 3.3. AT-RvD1 Downregulates the Phosphorylation of Transcription Factors

**Figure 3:**
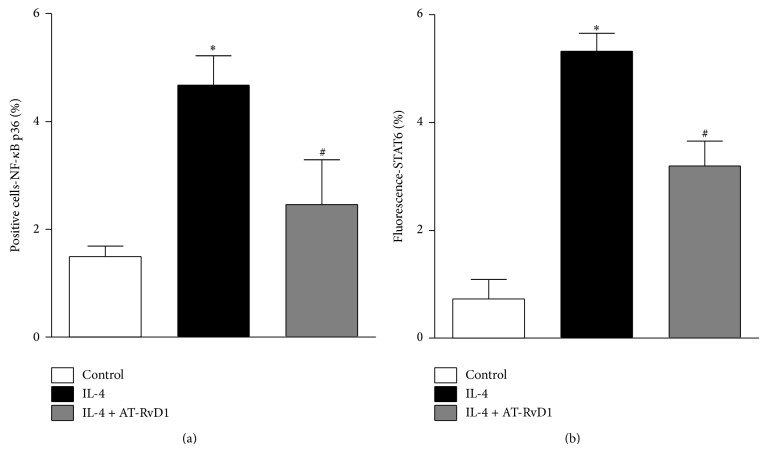
AT-RvD1 downregulates the NF-*κ*B (a) and STAT6 (b) pathways in bronchial epithelial cells stimulated with IL-4. BEAS-2B cells were stimulated with IL-4 (25 ng/mL) for 15 min in the presence or absence of AT-RvD1 (100 nM). The results are expressed as the arithmetic mean plus SEM from three independent experiments (*n* = 4). ^∗^ *P* < 0.05 versus control group; ^#^ *P* < 0.05 versus IL-4-treated group.

We next evaluated the effect of AT-RvD1 on the STAT6 and NF-*κ*B pathways. Signal transducer and activator of transcription 6 (STAT6) and nuclear factor kappa B (NF-*κ*B) have been demonstrated to regulate many pathologic features of asthma, and both are activated by IL-4 [26, 27]. As shown in Figures 3(a) and 3(b), IL-4 induced the significant phosphorylation of NF-*κ*B and STAT6 in cells compared to the control. Of note, AT-RvD1 significantly reduced cells expressing of NF-*κ*B (Figure 3(a)) and STAT6 (Figure 3(b)) when compared to cells treated only with IL-4.

### 3.4. AT-RvD1 Acts in Modulating the Expression of SOCS1 and SOCS3

**Figure 4:**
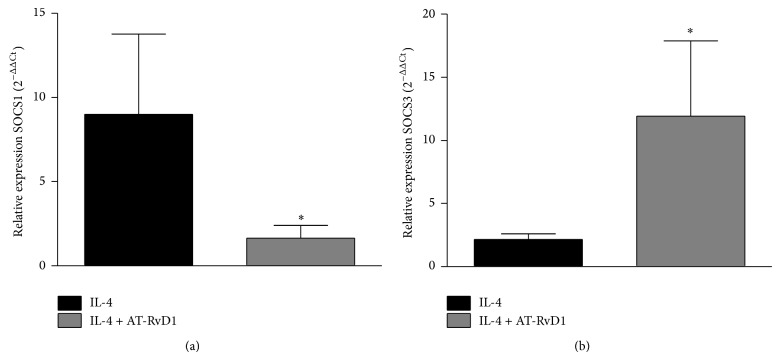
AT-RvD1 decreases SOCS1 (a) and increases SOCS3 (b) expression in bronchial epithelial cells stimulated with IL-4 (50 ng/mL). BEAS-2B cells were treated with AT-RvD1 (100 nM) 30 minutes before IL-4 stimulation. At 1 hour after stimulation, SOCS expression was quantified by qPCR. The results are expressed as the mean ± EPM (*n* = 4). ^∗^ *P* < 0.05 versus IL-4.

As the SOCS family is known to inhibit STAT signaling, we next evaluated the effect of AT-RvD1 on SOCS1 and SOCS3. In these experiments, the dose of 50 ng/mL was used for stimulation because the dose of 25 ng/mL did not induce the SOCSs expression (data not shown); this is in agreement with previous results [27]. The results showed that AT-RvD1 significantly reduced the expression of SOCS1 when compared with cells stimulated with IL-4 (Figure 4(a)); moreover, AT-RvD1 significantly increased SOCS3 expression (Figure 4(b)).

## 4. Discussion

IL-4 coordinates the Th2 immune response, which is associated with the pathophysiology of asthma. Interesting lipids mediators of resolution, such as AT-RvD1, demonstrate significant anti-inflammatory and proresolution effects in several experimental models. Here, we demonstrate for the first time the effect of AT-RvD1 in bronchial epithelial cells stimulated with IL-4. AT-RvD1 significantly reduced CCL2 and CXCL-8 production when compared to cells treated with IL-4. These effects are ALX/FPR2 receptor dependent and in part associated with the downregulation of STAT6 and NF-*κ*B pathways by AT-RvD1. Therefore, AT-RvD1 decreased SOCS1 and increased SOCS3 expression, which play critical roles in lymphocyte differentiation, maturation, and function. These results suggest that AT-RvD1 can modulate the innate and adaptive immune responses of asthma and other diseases, but further studies are needed for confirmation.

IL-4 is the major factor in the differentiation of the Th2-type immune response and blocks the differentiation of Th1 cells by indirect inhibiting interferon-*γ* (IFN-*γ*) [28]. Bronchial epithelial cells express IL-4 receptor (IL-4R), and IL-4 induces the production of chemokines such as CCL2 and CXCL-8, among other inflammatory parameters [7, 22, 24–26]. CCL2, also known as monocyte chemotactic protein-1 (MCP-1), is a potent chemotactic for monocytes and is produced constitutively or after stimulation in various cell types, including bronchial epithelial cells [27]. Indeed, CCL2 is chemotactic to monocytes/macrophages, basophils, eosinophils, and Th2 cells. In addition, CCL2 is involved in the polarization of Th2 cells and therefore is associated with the pathogenesis of allergic inflammatory diseases, such as asthma [29, 30]. Most patients with asthma have symptoms that are readily controllable by standard asthma therapies [2]. However, 5–10% of asthmatic individuals have poorly controlled disease with frequent exacerbations or symptoms that are refractory to current therapy [2, 3]. Distinct from the airway inflammation of stable asthma, which has been attributed to ongoing Th2-mediated inflammation, with a predominance of eosinophils and lymphocytes, there is increasing evidence to suggest that the increased inflammation in asthma exacerbation is under different regulation [31]. In addition to the eosinophils and lymphocytes that predominate in Th2-type inflammation, asthma exacerbations are notable for a neutrophil-enriched inflammatory response, which in some cases is the principal cellular infiltrate. Neutrophils are the major inflammatory cell in the airways of individuals dying within several hours of an asthma attack and are found in increasing numbers in patients dying of status asthmaticus [32]. Their numbers are increased in the sputum and bronchial washings of patients intubated for status asthmaticus [33–35]. There are several chemoattractants for neutrophils, such as the CXCL-8 [36] and the lipid mediator leukotriene B4 (LTB_4_) [37]. CXCL-8 is a chemokine that is mainly involved in the recruitment of neutrophils and exerts this effect by binding to two cell surface receptors, chemokine receptors CXCR1 and CXCR2 [36]. In addition to neutrophils, CXCL-8 may also recruit B and T lymphocytes, NK cells, and dendritic cells [38–40]. In addition, CXCL-8 induces the degranulation of neutrophils, basophils, and macrophages [41].

LTB_4_ and proinflammatory lipids mediators are well known to play important roles in asthma [42], but not all lipid mediators are associated with inflammation. For example, lipoxins and resolvins and their epimers are lipids mediators generated during the resolution phase and demonstrate significant anti-inflammatory and proresolution effects [43, 44]. In a previous study, our group demonstrated that AT-RvD1 markedly decreased airway eosinophilia and mucus metaplasia, in part by decreasing IL-5 and IkB*α* degradation in allergen-sensitized and challenged mice. In addition, AT-RvD1 significantly enhanced the macrophage phagocytosis of IgG-OVA-coated beads in vitro and in vivo, a new proresolving mechanism for the clearance of allergens from the airways [20]. In the present work, AT-RvD1 significantly reduced CCL2 and CXCL-8 production in bronchial epithelial cells when compared to cells stimulated with IL-4, demonstrating the potential to reduce both neutrophilic and eosinophilic inflammation in asthma.

AT-RvD1 can serve as an agonist for the ALX/FPR2 receptor to transduce, in part, its proresolution action [45–48]. The ALX/FPR2 receptor is broadly expressed in airway epithelial cells and alveolar macrophages and is dynamically regulated during allergic airway responses, leading to decreased receptor abundance [20, 49]. These changes are similar to those observed in human asthma [50]. We demonstrated that the inhibitory effect of AT-RvD1 on chemokine production by BEAS-2B cells stimulated with IL-4 is ALX/FPR2 receptor dependent, because the antagonist of this receptor reversed its effects.

Several transcription factors have also been implicated in the inflammatory process of asthma, including STAT6 and NF-*κ*B [51–54]. STAT6 has been demonstrated to regulate many pathologic features of lung inflammatory responses, including Th2 cell differentiation, airway eosinophilia, epithelial mucus production, and smooth muscle changes [55, 56]. NF-*κ*B controls the expression of some relevant genes encoding chemokines (CCL11, CXCL-8), cytokines (IL-5), and adhesion molecules (P-selectin) involved in airway eosinophilic and/or neutrophilic inflammation [57–60]. AT-RvD1 demonstrated a significant effect in reducing the phosphorylation of both STAT6 and NF-*κ*B in BEAS-2B cells stimulated with IL-4. The downregulation of NF-*κ*B by AT-RvD1 is in agreement with a previous study by our group [19, 20]; however, the present study is the first to demonstrate STAT6 modulation by AT-RvD1.

The JAK/STAT pathways have a pivotal role in the differentiation of helper T cells. The SOCS family, induced by cytokine stimulation, inhibits STAT signaling [59, 60]. SOCS1 has been shown to be a critical negative regulator of IFN-*γ* and consequently of the Th1 immune response [61]. SOCS3 promotes Th2 differentiation by blocking STAT4 signaling. However, the removal of SOCS3 from T cells inhibits Th1 and Th2 responses [62, 63]. In addition, SOCS3 blocks STAT3 signaling and consequently inhibits Th17 polarization [64]. IL-17 plays an important role in the development of severe asthma due to induced neutrophilic inflammation [65, 66]. Therefore, the inhibition of Th17 cell differentiation or IL-17 production could be beneficial for controlling severe asthma. SOCS plays an important role in the modulation of inflammation and is critical due to its broad spectrum of signaling events. However, the role of SOCS in bronchial epithelial cells is not clear. In our experiments, IL-4 increased both SOCS1 and SOCS3 expression, with SOCS1 showing higher expression, whereas AT-RvD1 decreased SOCS1 and increased SOCS3 expression compared to cells stimulated with IL-4. Thus, it is possible that SOCS1 inhibition and SOCS3 induction, involved in Th1 and Th17 immune responses, respectively, by AT-RvD1 may also negatively regulate JAK/STAT signaling pathways in BEAS-2B cells. However, additional studies are needed to test this hypothesis. Taken together, the results suggested that AT-RvD1 has a potential to modulate the immune response in both stable and severe asthma.

## 5. Conclusion

In conclusion, our results demonstrate that AT-RvD1 modulates the activation of bronchial epithelial cells induced by IL-4. AT-RvD1, via the ALX/FPR2 receptor, decreased CCL2 and CXCL-8 production and downregulated the NF-*κ*B and STAT6 pathways. In addition, AT-RvD1 decreased SOCS1 and increased SOCS3 expression. Together, these results suggest that AT-RvD1 has the potential to control airway inflammation.

## References

[1] Kelly-Welch A. E., Hanson E. M., Boothby M. R., Keegan A. D. (2003). Interleukin-4 and interleukin-13 signaling connections maps. *Science*.

[2] Fanta C. H. (2009). Asthma. *The New England Journal of Medicine*.

[3] Busse W. W., Lemanske R. F. (2001). Asthma. *The New England Journal of Medicine*.

[4] Lloyd C. M., Hessel E. M. (2010). Functions of T cells in asthma: more than just T_H_2 cells. *Nature Reviews Immunology*.

[5] Hammad H., Lambrecht B. N. (2008). Dendritic cells and epithelial cells: linking innate and adaptive immunity in asthma. *Nature Reviews Immunology*.

[6] Sha Q., Truong-Tran A. Q., Plitt J. R., Beck L. A., Schleimer R. P. (2004). Activation of airway epithelial cells by toll-like receptor agonists. *American Journal of Respiratory Cell and Molecular Biology*.

[7] Kato A., Favoreto S., Avila P. C., Schleimer R. P. (2007). TLR3- and Th2 cytokine-dependent production of thymic stromal lymphopoietin in human airway epithelial cells. *The Journal of Immunology*.

[8] Kauffman H. F. (2006). Innate immune responses to environmental allergens. *Clinical Reviews in Allergy and Immunology*.

[9] Bilyk N., Holt P. G. (1993). Inhibition of the immunosuppressive activity of resident pulmonary alveolar macrophages by granulocyte/macrophage colony-stimulating factor. *Journal of Experimental Medicine*.

[10] Ebeling C., Lam T., Gordon J. R., Hollenberg M. D., Vliagoftis H. (2007). Proteinase-activated receptor-2 promotes allergic sensitization to an inhaled antigen through a TNF-mediated pathway. *The Journal of Immunology*.

[11] Kiss A., Montes M., Susarla S. (2007). A new mechanism regulating the initiation of allergic airway inflammation. *Journal of Allergy and Clinical Immunology*.

[12] Stumbles P. A., Strickland D. H., Pimm C. L. (2001). Regulation of dendritic cell recruitment into resting and inflamed airway epithelium: use of alternative chemokine receptors as a function of inducing stimulus. *Journal of Immunology*.

[13] Lilly C. M., Nakamura H., Kesselman H. (1997). Expression of eotaxin by human lung epithelial cells: induction by cytokines and inhibition by glucocorticoids. *The Journal of Clinical Investigation*.

[14] Reibman J., Hsu Y., Chen L. C., Bleck B., Gordon T. (2003). Airway epithelial cells release MIP-3*α*/CCL20 in response to cytokines and ambient particulate matter. *American Journal of Respiratory Cell and Molecular Biology*.

[15] Webb D. C., Cai Y., Matthaei K. I., Foster P. S. (2007). Comparative roles of IL-4, IL-13, and IL-4R*α* in dendritic cell maturation and CD4^+^ Th2 cell function. *The Journal of Immunology*.

[16] Wen F. Q., Kohyama T., Liu X. (2002). Interleukin-4- and interleukin-13-enhanced transforming growth factor-*β*2 production in cultured human bronchial epithelial cells is attenuated by interferon-*γ*. *The American Journal of Respiratory Cell and Molecular Biology*.

[17] Ip W. K., Wong C. K., Lam C. W. K. (2006). Interleukin (IL)-4 and IL-13 up-regulate monocyte chemoattractant protein-1 expression in human bronchial epithelial cells: Involvement of p38 mitogen-activated protein kinase, extracellular signal-regulated kinase 1/2 and Janus kinase-2 but not c-Jun NH2-terminal kinase 1/2 signalling pathways. *Clinical and Experimental Immunology*.

[18] Serhan C. N., Hong S., Gronert K. (2002). Resolvins: a family of bioactive products of omega-3 fatty acid transformation circuits initiated by aspirin treatment that counter proinflammation signals. *The Journal of Experimental Medicine*.

[19] Eickmeier O., Seki H., Haworth O. (2013). Aspirin-triggered resolvin D1 reduces mucosal inflammation and promotes resolution in a murine model of acute lung injury. *Mucosal Immunology*.

[20] Rogerio A. P., Haworth O., Croze R. (2012). Resolvin D1 and aspirin-triggered resolvin D1 promote resolution of allergic airways responses. *The Journal of Immunology*.

[21] Bonnans C., Gras D., Chavis C. (2007). Synthesis and anti-inflammatory effect of lipoxins in human airway epithelial cells. *Biomedicine and Pharmacotherapy*.

[22] Cao J., Wong C. K., Yin Y., Lam C. W. K. (2010). Activation of human bronchial epithelial cells by inflammatory cytokines IL-27 and TNF-*α*: Implications for immunopathophysiology of airway inflammation. *Journal of Cellular Physiology*.

[23] Livak K. J., Schmittgen T. D. (2001). Analysis of relative gene expression data using real-time quantitative PCR and the 2-ΔΔCT method. *Methods*.

[24] Porter J. C., Hall A. (2009). Epithelial ICAM-1 and ICAM-2 regulate the egression of human T cells across the bronchial epithelium. *FASEB Journal*.

[25] Thompson A. B., Robbins R. A., Romberger D. J. (1995). Immunological functions of the pulmonary epithelium. *European Respiratory Journal*.

[26] Wen F.-Q., Kohyama T., Liu X. (2002). Interleukin-4- and interleukin-13-enhanced transforming growth factor-*β*2 production in cultured human bronchial epithelial cells is attenuated by interferon-*γ*. *American Journal of Respiratory Cell and Molecular Biology*.

[27] Wu D., Tan W., Zhang Q., Zhang X., Song H. (2014). Effects of ozone exposure mediated by BEAS-2B cells on T cells activation: a possible link between environment and asthma. *Asian Pacific Journal of Allergy and Immunology*.

[28] Nakamura T., Kamogawa Y., Bottomly K., Flavell R. A. (1997). Polarization of IL-4- and IFN-*γ*-producing CD4 + T cells following activation of naive CD4+ T cells. *The Journal of Immunology*.

[29] Hebenstreit D., Luft P., Schmiedlechner A. (2003). IL-4 and IL-13 induce *SOCS-1* gene expression in A549 cells by three functional STAT6-binding motifs located upstream of the transcription initiation site. *The Journal of Immunology*.

[30] Gu L., Tseng S., Horner R. M., Tam C., Loda M., Rollins B. J. (2000). Control of T_H_2 polarization by the chemokine monocyte chemoattractant protein-1. *Nature*.

[31] (2000). Proceedings of the ATS workshop on refractory asthma: current understanding, recommendations, and unanswered questions. American Thoracic Society. *American Journal of Respiratory and Critical Care Medicine*.

[32] Sur S., Crotty T. B., Kephart G. M. (1993). Sudden-onset fatal asthma: a distinct entity with few eosinophils and relatively more neutrophils in the airway submucosa?. *American Review of Respiratory Disease*.

[33] Fahy J. V., Kim K. W., Liu J., Boushey H. A. (1995). Prominent neutrophilic inflammation in sputum from subjects with asthma exacerbation. *Journal of Allergy and Clinical Immunology*.

[34] Lamblin C., Gosset P., Tillie-Leblond I. (1998). Bronchial neutrophilia in patients with noninfectious status asthmaticus. *American Journal of Respiratory and Critical Care Medicine*.

[35] Twaddell S. H., Gibson P. G., Carty K., Woolley K. L., Henry R. L. (1996). Assessment of airway inflammation in children with acute asthma using induced sputum. *European Respiratory Journal*.

[36] Konrad F. M., Reutershan J. (2012). CXCR2 in acute lung injury. *Mediators of Inflammation*.

[37] Chung K. F. (2005). Inflammatory mediators in chronic obstructive pulmonary disease. *Current Drug Targets: Inflammation and Allergy*.

[38] Allen T. C., Kurdowska A. (2014). Interleukin 8 and acute lung injury. *Archives of Pathology and Laboratory Medicine*.

[39] Todorović-Raković N., Milovanović J. (2013). Interleukin-8 in breast cancer progression. *Journal of Interferon and Cytokine Research*.

[40] Dhooghe B., Noël S., Huaux F., Leal T. (2014). Lung inflammation in cystic fibrosis: pathogenesis and novel therapies. *Clinical Biochemistry*.

[41] Stuart M. J., Baune B. T. (2014). Chemokines and chemokine receptors in mood disorders, schizophrenia, and cognitive impairment: a systematic review of biomarker studies. *Neuroscience & Biobehavioral Reviews*.

[42] Chung E. H., Jia Y., Ohnishi H. (2014). Leukotriene B4 receptor 1 is differentially expressed on peripheral T cells of steroid-sensitive and -resistant asthmatics. *Annals of Allergy, Asthma and Immunology*.

[43] Serhan C. N. (2010). Novel lipid mediators and resolution mechanisms in acute inflammation: to resolve or not?. *American Journal of Pathology*.

[44] Russell C. D., Schwarze J. (2014). The role of pro-resolution lipid mediators in infectious disease. *Immunology*.

[45] Krishnamoorthy S., Recchiuti A., Chiang N. (2010). Resolvin D1 binds human phagocytes with evidence for proresolving receptors. *Proceedings of the National Academy of Sciences of the United States of America*.

[46] Norling L. V., Dalli J., Flower R. J., Serhan C. N., Perretti M. (2012). Resolvin D1 limits polymorphonuclear leukocyte recruitment to inflammatory loci: receptor-dependent actions. *Arteriosclerosis, Thrombosis, and Vascular Biology*.

[47] Krishnamoorthy S., Recchiuti A., Chiang N., Fredman G., Serhan C. N. (2012). Resolvin D1 receptor stereoselectivity and regulation of inflammation and proresolving MicroRNAs. *American Journal of Pathology*.

[48] Perretti M., Chiang N., La M. (2002). Endogenous lipid- and peptide-derived anti-inflammatory pathways generated with glucocorticoid and aspirin treatment activate the lipoxin A4 receptor. *Nature Medicine*.

[49] Wang B., Gong X., Wan J.-Y. (2011). Resolvin D1 protects mice from LPS-induced acute lung injury. *Pulmonary Pharmacology and Therapeutics*.

[50] Planagumà A., Kazani S., Marigowda G. (2008). Airway lipoxin A4 generation and lipoxin A4 receptor expression are decreased in severe asthma. *The American Journal of Respiratory and Critical Care Medicine*.

[51] Barnes P. J., Adcock I. M. (2009). Glucocorticoid resistance in inflammatory diseases. *The Lancet*.

[52] Guo Q., Xu Y., Zhang Z. (2005). Role of activator protein-1 in the transcription of interleukin-5 gene regulated by protein kinase C signal in asthmatic human T lymphocytes. *Journal of Huazhong University of Science and Technology*.

[53] Nakamura Y., Hoshino M. (2005). TH2 cytokines and associated transcription factors as therapeutic targets in asthma. *Current Drug Targets: Inflammation and Allergy*.

[54] Poynter M. E., Cloots R., van Woerkom T. (2004). NF-*κ*B activation in airways modulates allergic inflammation but not hyperresponsiveness. *Journal of Immunology*.

[55] Iwata A., Kawashima S., Kobayashi M. (2014). T_h_2-type inflammation instructs inflammatory dendritic cells to induce airway hyperreactivity. *International Immunology*.

[56] Fu Q., Wang J., Ma Z., Ma S. (2014). Anti-asthmatic effects of matrine in a mouse model of allergic asthma. *Fitoterapia*.

[57] Rothenberg M. E., Luster A. D., Leder P. (1995). Murine eotaxin: an eosinophil chemoattractant inducible in endothelial cells and in interleukin 4-induced tumor suppression. *Proceedings of the National Academy of Sciences of the United States of America*.

[58] Anrather J., Csizmadia V., Brostjan C., Soares M. P., Bach F. H., Winkler H. (1997). Inhibition of bovine endothelial cell activation in vitro by regulated expression of a transdominant inhibitor of NF-*κ*B. *The Journal of Clinical Investigation*.

[59] Yang L., Cohn L., Zhang D.-H., Homer R., Ray A., Ray P. (1998). Essential role of nuclear factor *κ*B in the induction of eosinophilia in allergic airway inflammation. *The Journal of Experimental Medicine*.

[60] Serio K. J., Reddy K. V., Bigby T. D. (2005). Lipopolysaccharide induces 5-lipoxygenase-activating protein gene expression in THP-1 cells via a NF-*κ*B and C/EBP-mediated mechanism. *The American Journal of Physiology—Cell Physiology*.

[61] Linossi E. M., Babon J. J., Hilton D. J., Nicholson S. E. (2013). Suppression of cytokine signaling: the SOCS perspective. *Cytokine and Growth Factor Reviews*.

[62] Inagaki-Ohara K., Kondo T., Ito M., Yoshimura A. (2013). SOCS, inflammation, and cancer. *JAKSTAT*.

[63] Carow B., Rottenberg M. E. (2014). SOCS_3_: a major regulator of infection and inflammation. *Frontiers in Immunology*.

[64] Palmer D. C., Restifo N. P. (2009). Suppressors of cytokine signaling (SOCS) in T cell differentiation, maturation, and function. *Trends in Immunology*.

[65] Li Y., Hua S. (2014). Mechanisms of pathogenesis in allergic asthma: role of interleukin-23. *Respirology*.

[66] Morishima Y., Ano S., Ishii Y. (2013). Th17-associated cytokines as a therapeutic target for steroid-insensitive asthma. *Clinical and Developmental Immunology*.

